# Looking Through a Different Lens: Patient Satisfaction With Telemedicine in Delivering Pediatric Fracture Care

**DOI:** 10.5435/JAAOSGlobal-D-19-00100

**Published:** 2019-09-23

**Authors:** Neha Sinha, Max Cornell, Benjamin Wheatley, Nicole Munley, Mark Seeley

**Affiliations:** From the Department of Orthopaedic Surgery, Geisinger Medical Center, Danville, PA (Dr. Sinha, Mr. Cornell, Ms. Munley, and Dr. Seeley) and Department of Mechanical Engineering, Bucknell University, Lewisburg, PA (Dr. Wheatley and Ms. Munley).

## Abstract

Telemedicine may transform health care by overcoming geographical and travel-associated barriers to patient care. This study assesses patient satisfaction with telemedicine for fracture care.

**Methods:**

Two groups of patients were compared from suburban/rural Pennsylvania. One group reported to a regional medical center for real-time video consultation with a pediatric orthopaedic surgeon facilitated by a physician's assistant. The other group underwent conventional outpatient clinic visits at a tertiary care hospital. The distance between the tertiary care hospital and the regional medical center was 69 miles. New or follow-up fracture patients not living in the vicinity of either medical center were included. A satisfaction survey and questionnaire were administered to both groups at the end of their visit.

**Results:**

One hundred sixty-seven patients returned the questionnaires (66 conventional and 101 telemedicine). Telemedicine visits decreased indirect and direct costs (*P* = 0.032). Travel costs and travel times were lower (*P* < 0.001) in the telemedicine group. Patient satisfaction was similar. Only 8 of 101 patients in the telemedicine cohort preferred their next visit to be a conventional follow-up.

**Discussion:**

Utilization of video consultation and trained physician assistants to provide pediatric orthopaedic care across suburban/rural areas can increase pediatric orthopaedic surgeon access and decrease travel costs while maintaining patient satisfaction.

Telemedicine provides an exciting opportunity for patients to access healthcare workers and subspecialists to reach out to patients in remote and rural areas. The financial burden associated with travel and the distances that have to be covered to book an appointment with a physician often act as barriers to care, and this cost is often amplified when seeking care from a subspecialist.^1,2^ Although a large body of literature illustrates the role telehealth plays in decreasing a patient's travel time, reducing work missed, decreasing costs associated with traveling to an appointment, and expanding physician reach in various specialties,^3–5^ the application of telehealth in pediatric orthopaedics is still in its infancy.

The primary outcome of the study was to assess patient satisfaction in using telecommunication for fracture care.

## Methods

The study was designed as a quality improvement project to analyze the quality of telemedicine consultations in an outpatient clinic at a regional medical center as compared to standard consultation in an orthopaedic outpatient clinic at a tertiary care center. This study was carried out in rural central Pennsylvania. The distance between the medical centers was 69 miles.

For the purpose of this study, telemedicine consultation refers to a real-time voice and video session using Skype for Business on a tablet or PC with a pediatric orthopaedic physician's assistant (PA) at the regional medical center connecting to a pediatric orthopaedic surgeon at the tertiary hospital using a Health Insurance Portability and Accountability Act-compliant secured platform.

Two patient groups were compared. The first group included pediatric patients younger than 18 years of age (along with their parents or legal guardians) who used telemedicine with a trained orthopaedic PA present at the regional medical center. The second group included pediatric patients younger than 18 years of age (along with their parents or legal guardians) who were seen at the same attending physician's outpatient pediatric orthopaedic clinic at the tertiary hospital. The study was carried out over a 4-month period with all patients receiving a satisfaction survey at the end of the clinic visit for both sites (Supplemental Table 1, http://links.lww.com/JG9/A54). For the purpose of the study, being a new/referred or follow-up fracture patient was the inclusion criterion. Pediatric patients with complex congenital syndromes, non–fracture-related diagnoses, and developmental delay or neuromuscular diseases were excluded. In addition, participants living in the immediate vicinity of the medical centers were excluded from the study.

Participants in both groups were provided the option of teleconsultation at the time of scheduling their appointments. After screening for inclusion and exclusion criteria, participants were enrolled in the study. This was done by a trained PA in the first group, who also performed the physical examination of the patient, with management of care being directed by the pediatric orthopaedic surgeon. The participants in the second group were recruited by the same pediatric orthopaedic surgeon in the tertiary hospital's outpatient clinic. At the end of their visit, participants in both groups were invited to complete a survey. This was not a validated survey; however, it mimicked previously used satisfaction surveys in the outpatient clinic.

Electronic health records of the participants were reviewed, and demographic data including age and sex and details of diagnosis were analyzed.

Statistical analysis was performed on all questions from the survey (Supplemental Tables 1 and 2, http://links.lww.com/JG9/A54 and http://links.lww.com/JG9/A55). Minitab 17 was used for all statistical analyses. For binary data (yes/no), the Fisher exact test was performed with a significance of *P* < 0.05 to compare the survey results between patients who visited the clinic and patients who met with the physician through telemedicine. For all other data, a Mann-Whitney *U* test with a significance of *P* < 0.05 was used for the same group comparisons. This test was used because of the assumption of nonparametric data for all Likert scale data and travel measurement groupings.

A final questionnaire section included an opportunity for patients' guardians to record their future consultation preference. These questions analyzed whether telemedicine participants would prefer a visit to the tertiary hospital and whether in-clinic participants were familiar with telemedicine. In addition, the following three questions were asked:

(1) Instead of a telemedicine clinic consultation would you have rather just met with an advanced practitioner (example: nurse, physician assistance, or nurse practitioner)?
(2) Would you be willing to participate in a future telemedicine consultation?
(3) Which would you prefer: telemedicine consultation or physician on site?

## Results

In total, 167 patients meeting the inclusion and exclusion criteria completed the questionnaire, 66 patients from the tertiary hospital and 101 patients from the telemedicine site. The survey response rate at the remote telemedicine site was 88% with 14 surveys needing to be excluded: 2 for incomplete data and 12 for non–fracture-related issues. The clinic site survey response rate was 77% with 28 surveys being excluded: 3 for incomplete data and 25 for non–fracture-related issues. Patient demographics for each group were analyzed (Table 1). In comparison with in-clinic patient and guardian experience, telemedicine consultation resulted in less cost (both direct and indirect) to the patient/guardian for follow-up appointments (Table 2). This is observed through a statistical analysis of indirect costs, where patients' guardians were less likely to miss work for a telemedicine consultation versus in-clinic (*P* = 0.032). Travel time was also decreased (*P* = 0.0021) in the telemedicine group. In a measurement of direct cost, telemedicine was less likely to result in travel cost in comparison with in-clinic appointments (*P* < 0.001). No statistical difference was observed in wait time between groups, but 11% of in-clinic patients had to wait more than 45 minutes (7 of 66) versus zero telemedicine patients (zero of 101). Similarly, travel distance was not found to be statistically different, but 18% of in-clinic patients had to travel more than 75 miles (12 of 66), whereas this was the case for only 4% (4 of 101) of telemedicine patients.

**Table 1:**
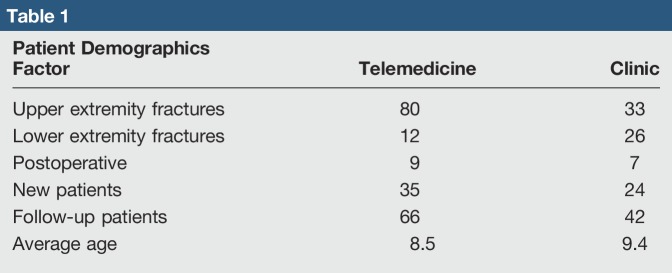
Patient Demographics

**Table 2:**
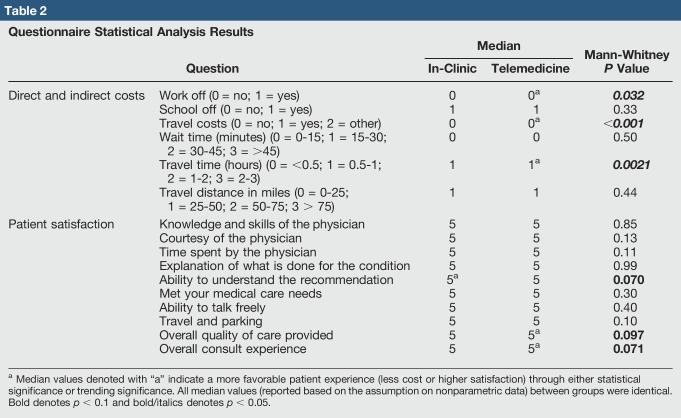
Questionnaire Statistical Analysis Results

In general, patients who received care through telemedicine were pleased with the overall experience in comparison with in-clinic patients (Table 2). For seven of the 10 patient satisfaction questions, no statistically significant differences were observed between groups. No significant difference was found in the overall quality of care provided (*P* = 0.097) and overall consult experience (*P* = 0.071). The in-clinic patients did report an increase in the ability to understand the physician recommendation in comparison with telemedicine patients, but this was not found to be significant (*P* = 0.070).

The average time taken for a telemedicine visit was 3 minutes 45 seconds. This represents the time spent by the physician with the patient. The PA spent much more time with the family. This time information was captured from the call logs of Skype for Business.

The final patient response section—future consultation preference—exhibits the willingness of patients who received a consultation through telemedicine to continue using a telemedicine approach (Table 3). Of the 101 participants in the telemedicine cohort, only 8 were found to prefer a future in-clinic visit and travel to a tertiary care center as compared to 38 who preferred telemedicine consultation (55 participants did not have a preference between the two modalities of patient care). Patients who visited the clinic appear less willing to partake in, or prefer, a telemedicine consultation.

**Table 3:**
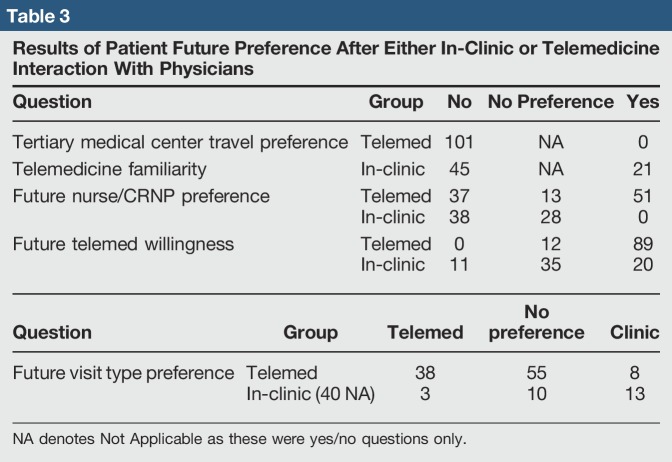
Results of Patient Future Preference After Either In-Clinic or Telemedicine Interaction With Physicians

When asked for any additional comment on the telemedicine experience, patients' guardians responded with only positive reflections:

It's a fabulous idea. No travel. Happy Mom.

Everything was well controlled.

I appreciated knowing my daughter was getting the expertise of Pediatric Orthopaedics without having to miss more school/work.

Thank you!

## Discussion

The primary goal of the study was to assess patients' (as well as parents' and legal guardians') satisfaction in using telecommunication for fracture care and to analyze various parameters that may be affecting patient satisfaction. An attempt was made to determine the financial implication of using telemedicine.

The present study is the first to report on patient cost, satisfaction, and future preference for in-clinic versus telemedicine pediatric orthopaedic consultations. The specific findings of the present study are a lower cost and no notable difference in patient satisfaction between telemedicine consultations and in-clinic consultations. We suggest that these two factors are linked because perceived quality of care may be a result of the saved costs—both indirect and direct—in telehealth versus in-clinic appointments. Specifically, less missed work (*P* = 0.032), less travel costs (*P* < 0.001), and less travel time (*P* = 0.021) were observed in our cohort. These findings suggest a telemedicine pediatric orthopaedic follow-up consultation is economically advantageous to the patient and just as satisfactory (if not more so) in comparison with an in-clinic appointment.

One weakness of the study is the potential for selection bias, given that patients self-opted to participate in a telemedicine visit. This study looks only at patients with fractures. However, it is possible that there may be more complex diagnoses which would require an orthopaedic surgeon to be present and telemedicine may not be an ideal option for delivering medical care in such situations. Thus, the results of this study may not be extrapolated to diagnoses other than fracture care.

Although patients' guardians were more likely to take time off from work for in-clinic visits versus telemedicine consultations (*P* = 0.032), time off from school was similar between the groups (*P* = 0.33). This may be the result of guardians who work part-time being able to schedule the telehealth meeting during nonworking hours because of the reduced travel time (*P* = 0.021). Future work is needed to validate this assumption and to determine the specific amount of time lost in school and/or at work, as an afternoon dedicated to telehealth suggests an advantage over a full day missed because of an in-clinic consultation. Furthermore, although the wait time between groups was not observed to be statistically significant (*P* = 0.50), 11% of in-clinic patients had to wait for 45 minutes or more in comparison with zero telemedicine patients. This may be because of the physician seeing the telemedicine patients concurrently with a busy outpatient clinic. It is probable that the patients had to wait longer during the outpatient clinic visits because of more complex patients being seen in the clinic as opposed to the telemedicine site. The average time taken for a telemedicine visit was 3 minutes 45 seconds. Although time information on the standard clinic visit is not available, it was felt that the “in-person” clinic took much longer. This perceived discrepancy was most likely secondary to the family already being seen by the PA at the telemedicine site and to the family being more accepting of a shorter duration during the video consultation.

This was the first instance of implementing telemedicine for pediatric orthopaedic care at our institute. The study was intended to function as a pilot phase to determine patient satisfaction with this modality of health care. Although our study supports lesser direct and indirect costs for the patients, an analysis of the time and economic burden to the physician was not undertaken. The cost of implementing telemedicine depends a lot on the kind of model that is used. The attending physician saw telemedicine patients during a very busy on-site clinic practice. Although this was more time consuming for the physician, it helped offset the patient load on the clinic infrastructure and resources. On average, the telemedicine clinics allowed an additional 12 to 16 patients to be seen by the orthopaedic provider in addition to the clinic schedules. Telemedicine allows better utilization of resources at different sites.

Proof of concept of telehealth in adult orthopaedics begun nearly two decades ago. Haukipuro et al^6^ found that videoconferencing between primary and secondary care may be used in orthopaedic patients and concluded that telemedicine can serve as a viable alternative to the conventional outpatient clinic visit. More recently, Buvik et al^4^ conducted a randomized controlled trial that established video-assisted consultations as a safe method of examination for selected adult orthopaedic patients. However, the use of teleconsultation in the highly specialized field of pediatric orthopaedics has been less commonly practiced and described. This may be because of several reasons such as parents preferring their children to see the physician in person and worries about the inferior quality of a telemedicine consult. In a review of their telemedicine program for pediatric orthopaedics, Ono and Lindsey^7^ observed carrying out physical examination to be a definite challenge because the quality of the physical examination was dependent on the capabilities of the examiner at the remote site. To combat this issue, the present study used trained PAs to perform physician-guided physical examinations on patients. Other adult orthopaedic telemedicine studies have used trained PAs and nurses to perform physician-guided physical examinations with good results.^4,5,6,7,8^

A retrospective audit of pediatric orthopaedic telehealth consultations performed in Australia aimed to determine which Australian children most commonly used telehealth services.^9^ The authors found patients with disabilities, such as cerebral palsy and intellectual disabilities, used telehealth consultations from pediatric orthopaedic surgeons most commonly, and they attributed this to the increased cost and inconvenience of transport in this patient cohort. Although our study did not include patients with developmental and congenital conditions, this population would likely reap tremendous benefits by introducing telemedicine for delivering health care by decreasing travel times because transportation in such cases can often prove to be a daunting and challenging experience. Also supporting the use of telehealth in pediatric surgical specialties, Shivji et al^10^ found great clinician and patient satisfaction with video consultation in a Canadian children's hospital and proposed telehealth for pediatric surgical services as an effective way to consult and follow up patients who live in remote areas. However, of 259 reported video consults, only 2 were orthopaedic in nature.

Certainly, the demographic and geographic distribution of the patient group from this study play a role in the reported satisfaction and associated visit costs. The population location of central/northeastern Pennsylvania provides a distribution of rural, suburban, and urban patients. The ability to implement telehealth clearly has a greater impact on those in remote and/or rural locations in comparison with densely populated regions where more health resources are readily available.^4^ In addition, of note is the unequal distribution of pediatric orthopaedic surgeons currently practicing in the United States.^11^ Pediatric orthopaedic surgeon density correlates with population density, which further supports the claim that less densely populated suburban and rural areas would see a greater benefit from a pediatric orthopaedics telehealth program than an urban area. Thus, it remains unclear how the results presented here may differ in a densely populated region; nonetheless, the relevance of this work is apparent for the large majority of the United States' mainland.

One potential difficulty in enacting the approach used in this study is the need of a specially trained PA. However, the approach of incorporating a trained PA on-site with telehealth patients provides not only an expert available for physical interactions with patients but also a skilled practitioner who is able to communicate between the remote physician and patient. Notably, although not statistically significant, the only potential advantage reported in this study for in-clinic visits—an increase in the ability of a patient/legal guardian to understand physician recommendations—exhibits the strong need to develop and maintain a clear line of communication between the patient and physician.

The reported median values for patient satisfaction from this study were identical for each question between the groups. We think this to be an indicator of the quality of care provided by the physician, PAs, and facilities because the median values for all patient satisfaction questions were five out of five. In addition, the median values for travel metrics were dominated largely by many patients who traveled a relatively short distance for care. Patients who traveled a certain distance for telemedicine meetings would have had to increase their travel time and distance to visit the clinic. This supports the notion that telemedicine is a less expensive and similarly satisfactory method for patient-physician interaction in comparison with in-clinic visits.

The results of this study have been able to prove that patient satisfaction with healthcare delivery was similar in both telemedicine and inpatient clinic cohorts. It is important to note that mid-levels such as PAs are trained medical providers and can practice without having an orthopaedic physician present, especially for comparatively straightforward cases such as fracture care. The idea behind using a PA for this study also stemmed from the confidence in their ability to perform orthopaedic examination. Physical examination was an important component of the fracture care visit. In addition, our remote sites do not have cast technicians, and we wanted to make sure that if a patient needed a cast that this would be placed at the same visit. The intention was to ensure that standard of care was not compromised while introducing telemedicine in our patient cohort. Promising results in the study have made it possible to consider expanding the practice of telemedicine with nursing staff and teaching them how to conduct an orthopaedic examination and place casts. This would also make it possible for the PAs to run their own clinics, thereby further increasing access to orthopaedic care and ensuring optimum utilization of resources. Another approach would be to introduce telemedicine in a primary physician's office, further increasing the availability of subspecialty care in remote areas.

State regulations and requirements on how to implement telemedicine are not uniform. Telemedicine continues to gain popularity as an emerging method of providing care. It would be prudent to remember that orthopaedics requires a familiarity with specific physical examination techniques and other clinic resources. We would recommend that any institution looking at implementing such a program proceed cautiously to ensure that the quality of care is not compromised.

In conclusion, the findings of this study highlight the positive impacts a pediatric orthopaedic telehealth program can have on pediatric fracture patients and their caregivers. Additional longitudinal studies are needed to analyze the difference in follow up of patients between telemedicine and inpatient clinic visits. Additional research exploring the role of telemedicine in treating pediatric patients with more complex orthopaedic conditions is warranted.

Although the described telemedicine program proved financially beneficial for the patients with minimal cost to the physician/hospital, more research into the cost benefit analysis is needed to ensure a telemedicine approach that is economically sustainable and can be run efficiently while maintaining a busy conventional practice. Widespread acceptance of this modality of health care can only occur if it proves to be cost effective not only to the patients but also to the physicians, the insurers, and the healthcare system. In addition, although our study showed a higher overall satisfaction among patients/parents and guardians in the telemedicine versus the in-clinic group, larger studies are needed to validate telemedicine consults as being noninferior to in-clinic visits with no reduction in the quality of patient care.

## Supplementary Material

SUPPLEMENTARY MATERIAL
