# Supplementary material for: Looking Through a Different Lens: Patient Satisfaction With Telemedicine in Delivering Pediatric Fracture Care
Source: J Am Acad Orthop Surg Glob Res Rev. 2019 Sep 23;3(9):e100. doi: 10.5435/JAAOSGlobal-D-19-00100 (PMC6860133; doi:10.5435/JAAOSGlobal-D-19-00100)
Supplement: SUPPLEMENTARY MATERIAL [file jg9-3-e100-s001.docx]

Supplemental Table 1. Questionnaire provided to all patients.

| **Question** | **Response Categories** |
| --- | --- |
| Patient category | New Patient or Follow up? |
| Employment status | Not Employed, Full Time, or Part Time |
| Did you have to take a day off from work for the appointment today? | Yes or No |
| Did your child have to take a day off from school for the appointment today? | Yes or No |
| Did you incur any travel costs? | Yes/No/Other |
| How long was your wait time before being seen today? | Minutes: 0-15, 15-30, 30-45, or 45+ |
| How long did it take you to get to clinic today? | Minutes: 0-30, 30-60, 60-120, 120-180, 180+ |
| How far did you travel to get to the clinic today? | Miles: 0-25, 25-50, 50-75, 75+ |
