# Supplementary material for: Looking Through a Different Lens: Patient Satisfaction With Telemedicine in Delivering Pediatric Fracture Care
Source: J Am Acad Orthop Surg Glob Res Rev. 2019 Sep 23;3(9):e100. doi: 10.5435/JAAOSGlobal-D-19-00100 (PMC6860133; doi:10.5435/JAAOSGlobal-D-19-00100)
Supplement: SUPPLEMENTARY MATERIAL [file jg9-3-e100-s002.docx]

Supplemental Table 2. Questionnaire for all patients with five-point Likert scale. The prompt for this table reads “How would you rate today’s clinic consultation on the factors listed below?”

| Question | Very Dissatisfied | Dissatisfied | Neutral | Satisfied | Very Satisfied |
| --- | --- | --- | --- | --- | --- |
| Knowledge and skills of the Geisinger physician | 1 | 2 | 3 | 4 | 5 |
| Courtesy of Geisinger physician | 1 | 2 | 3 | 4 | 5 |
| Time spent by the Geisinger physician | 1 | 2 | 3 | 4 | 5 |
| Explanation of what is being done for your medical condition | 1 | 2 | 3 | 4 | 5 |
| Ability to understand the recommendation made | 1 | 2 | 3 | 4 | 5 |
| Met your medical care needs | 1 | 2 | 3 | 4 | 5 |
| Ability to talk freely | 1 | 2 | 3 | 4 | 5 |
| Travel and parking | 1 | 2 | 3 | 4 | 5 |
| Overall quality of care provided | 1 | 2 | 3 | 4 | 5 |
